# Authentication of differential gene expression in oral squamous cell carcinoma using machine learning applications

**DOI:** 10.1186/s12903-021-01642-9

**Published:** 2021-05-29

**Authors:** Rian Pratama, Jae Joon Hwang, Ji Hye Lee, Giltae Song, Hae Ryoun Park

**Affiliations:** 1 School of Computer Science and Engineering, Pusan National University, 63 Busandaehak-Ro, Busan, 46241 Republic of Korea; 2 Department of Oral and Maxillofacial Radiology, School of Dentistry, Pusan National University, Dental Research Institute, Yangsan, 50610 Republic of Korea; 3 Department of Oral Pathology, School of Dentistry, Pusan National University, 49 Busandaehak-Ro, Yangsan, 50612 Republic of Korea; 4 Periodontal Disease Signaling Network Research Center, School of Dentistry, Pusan National University, Yangsan, 50612 Republic of Korea

**Keywords:** The cancer genome atlas, Oral squamous cell carcinoma, Convolutional neural network, Tumour classification, Diagnostic model

## Abstract

**Background:**

Recently, the possibility of tumour classification based on genetic data has been investigated. However, genetic datasets are difficult to handle because of their massive size and complexity of manipulation. In the present study, we examined the diagnostic performance of machine learning applications using imaging-based classifications of oral squamous cell carcinoma (OSCC) gene sets.

**Methods:**

RNA sequencing data from SCC tissues from various sites, including oral, non-oral head and neck, oesophageal, and cervical regions, were downloaded from The Cancer Genome Atlas (TCGA). The feature genes were extracted through a convolutional neural network (CNN) and machine learning, and the performance of each analysis was compared.

**Results:**

The ability of the machine learning analysis to classify OSCC tumours was excellent. However, the tool exhibited poorer performance in discriminating histopathologically dissimilar cancers derived from the same type of tissue than in differentiating cancers of the same histopathologic type with different tissue origins, revealing that the differential gene expression pattern is a more important factor than the histopathologic features for differentiating cancer types.

**Conclusion:**

The CNN-based diagnostic model and the visualisation methods using RNA sequencing data were useful for correctly categorising OSCC. The analysis showed differentially expressed genes in multiwise comparisons of various types of SCCs, such as *KCNA10*, *FOSL2*, and *PRDM16*, and extracted leader genes from pairwise comparisons were *FGF20*, *DLC1*, and *ZNF705D*.

**Supplementary Information:**

The online version contains supplementary material available at 10.1186/s12903-021-01642-9.

## Background

Traditionally, the choice of treatment modality for cancer primarily depends on the histopathologic diagnosis, and a definite diagnosis plays a major role in choosing a treatment strategy and determining the prognosis of the disease [1, 2]. Thus, though the importance of an accurate diagnosis of cancer is substantial, studies on the development of new classifications of tumour types are rare, and current classification schemes primarily depend on the morphologic characteristics of tumour cells and tissues [3, 4]. However, there are numerous examples of cancers with the same diagnostic classification (based on similar histopathologic features) having different responses to therapies. These differences result from alterations of the biological behaviour of cancer cells via genetic variations as well as environmental factors [5–7]. Thus, a diagnostic model that predicts the biological behaviour of cancers by considering their genetic characteristics rather than only morphologic characteristics needs to be developed.

Understanding the genetic heterogeneity of cancers can provide clues for increasing diagnostic accuracy and developing efficient biomarkers as well as improving treatment efficacy [8–11]. A massive amount of cancer tissue genomic data have been generated due to the development of next-generation sequencing methods with high efficiency and accuracy, and most of the data are housed by The Cancer Genome Atlas (TCGA) database [12–14]. Using RNA sequencing expression data, studies have attempted to find a diagnostic model that can efficiently and rapidly discriminate different tumours by simultaneously considering both genetic phenotypic features [15–17]. Due to the difficulty in manually interpreting genomic datasets, various machine learning methods that are trained on differentially expressed gene sets across tumours have been used to analyse and classify tumours based on tumour-specific gene expression [18]. However, using generic machine learning methods such as genetic algorithms yields high dimensionality of the genomic dataset, and innovative methods are continuously being developed to increase performance. Recently, Lyu et al. developed a more efficient method by applying convolutional neural network (CNN) image classification, which is a state-of-the-art method for solving classification problems [19]. In addition, deep learning methods exploiting image classification/recognition have been suggested, and these methods have displayed excellent performance as well as a low error rate [17, 20]. Using methods such as these, tumour classifications based on machine learning analysis of genetic data have been rapidly developed and tested. However, research to establish a classification system to diagnose oral squamous cell carcinoma (OSCC) using genetic data is rare.

OSCC, the most common cancer of the oral cavity, derives from the oral mucosa (which is lined by stratified squamous epithelium) and shares morphologic findings and histochemical features with SCC of other sites or tissues. All SCCs are diagnosed as the same type of disease due to similar histologic findings, but the gene expression of each SCC has not been compared. Therefore, it is not well understood whether the genetic features of SCCs of various tissues or organs are similar. Knowledge of the genetic features distinguishing OSCC from SCCs of other tissues might provide clues for differential diagnosis and the development of treatment paradigms. In the present study, we examined the diagnostic performance of machine learning applications using CNN image classification for OSCC samples. In addition, we investigated the differences in genetic features between OSCC and other types of SCC, which may provide a basic rationale for potential diagnostic models and the development of targeted therapy.

## Methods

### Data procurement

RNA sequencing data from samples of OSCC, non-oral head and neck SCC (HNSCC), oesophageal SCC, oesophageal adenocarcinoma, and cervical SCC were downloaded from the TCGA database (http://cancergenome.nih.gov/). The platforms used, experiment types, and numbers of samples analysed for mRNA expression for each cancer are shown in Additional file 1: Table S1.

### Computational analysis

**Fig. 1:**
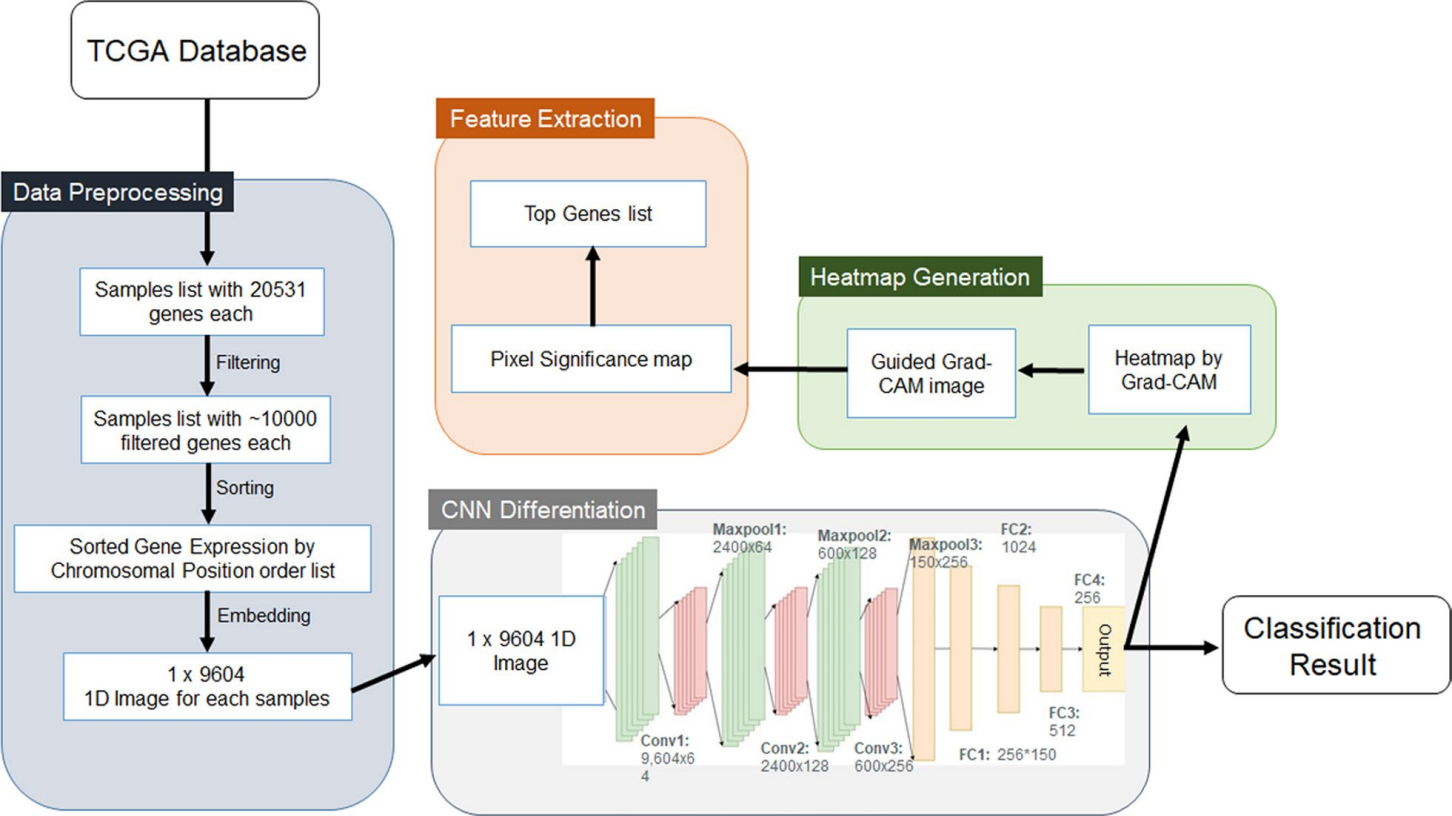
The workflow of this study

The workflow of our study is shown in Fig. 1. The feature extraction for differentiating SCCs based on gene expression was performed using the protocol in reference with a few modifications and consists of 4 steps: (1) data preprocessing, ((2) differentiation using CNN [21, 22], (3) heatmap generation, and ((4) feature extraction. The 4 steps are briefly explained below, and the detailed methods for CNN and heatmap generation are described in the following sections.

#### Data preprocessing

To cope with high-dimensional expression data, the RNA sequencing data of the TCGA dataset were embedded into 1-D images. Briefly, the normalised read count data of each gene with enormous values were transformed by using y = log2(x + 1), and noise data were filtered out by using a variance threshold of 1. Then, the data were embedded into 1-D images.

#### Differentiation

The differentiation step used the CNN model to process the 1-D images output from the preprocessing step. The training and testing were performed using a 10-fold cross validation method.

#### Heatmap generation

The class activation map method was used to visualise what the CNN had learned and heatmap images representing the contribution of each gene were produced from the image input.

#### Feature extraction

Since each pixel corresponds to one gene, feature extraction methods take the most important pixels with the most intensity within the image and categorise the corresponding represented genes into a list of dominant genes.

### CNN analysis

A CNN model [21, 22], which has multiple layers consisting of three convolutional layers and four fully connected layers, was implemented in this experiment. The first convolutional layer ‘conv1’ contained 64 different filters, while the second convolutional layer ‘conv2’ and the third convolutional layer ‘conv3’ contained 128 and 256 filters, respectively. Both a max-pooling layer and a batch-normalisation layer immediately followed each convolutional layer. A drop-out layer was added before entering the fully connected layer, and the drop-out rate was 25%. The sizes of the three fully connected layers were 36,864, 1,024, 512, and 256. We chose the cross-entropy method as a loss function and the Adam optimiser to update the weights. We used a tenfold cross validation method to train the CNN and to test the performance.

### CNN visualisation

Because we needed to extract features learned through CNN analysis, we employed the class activation map (CAM) method to visualise the learned features from the CNN output. The CAM method helps understand what regions of an input image influence the CNN’s output prediction. The technique relies on the generation of a heatmap for visualisation, which highlights pixels of the image that trigger the model to associate the image with a particular class. In this experiment, since each pixel represented a corresponding gene, the CAM method could visualise which gene was dominant for each type of cancer.

## Results

### The diagnostic tool using machine learning computational analysis exhibited excellent performance in the diagnosis of OSCC but had limited utility differentiating OSCC from non-oral HNSCC

**Fig. 2:**
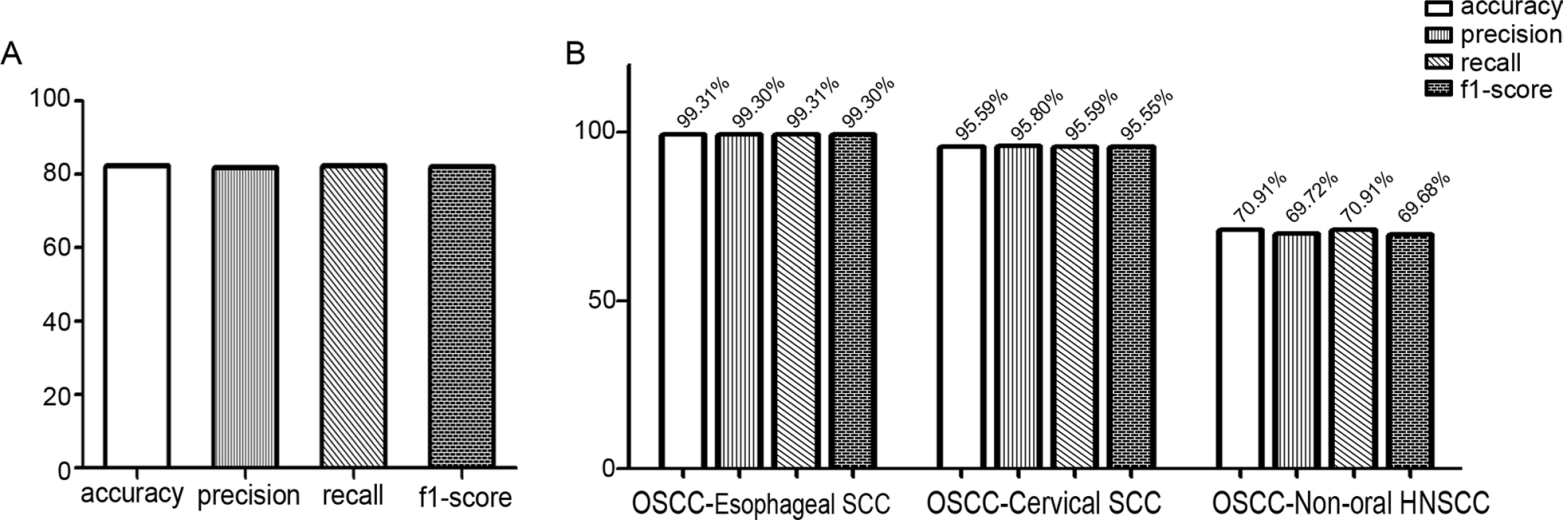
Performances of our method which compared various types of squamous cell carcinoma (SCC) genetic set in multiwise fashion (**A**) and compared individually oral SCC with other SCC (**B**)

**Fig. 3:**
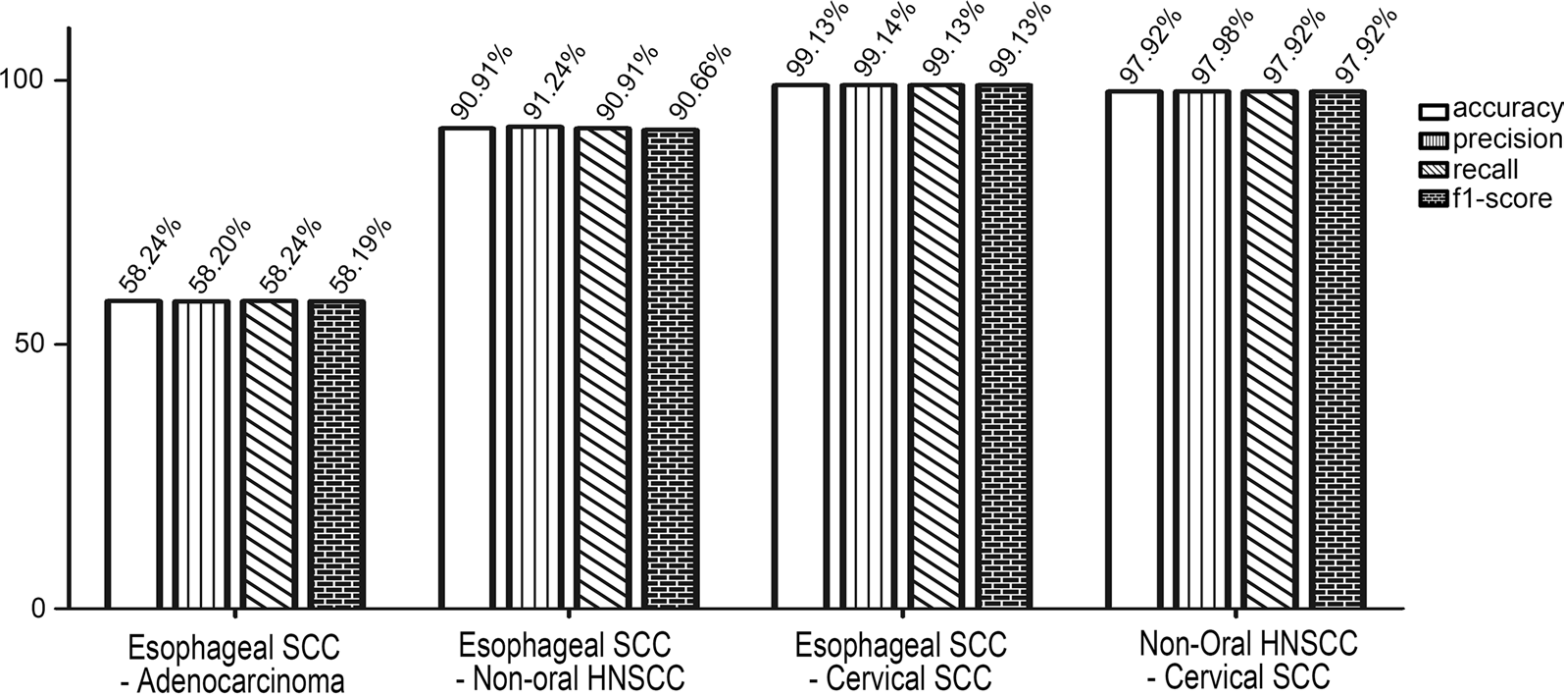
Performances of our method which compared non-oral head and neck SCC with oesophageal using SCC genetic set

To evaluate the performance of the tool in OSCC diagnosis, its performance capabilities, such as test recall, test precision, and test accuracy, were compared using the dataset containing OSCC, non-oral HNSCC, cervical SCC, and oesophageal SCC samples in a multiwise fashion. After repeated training using the dataset, the tool showed ~ 82% test precision and test accuracy in diagnosing SCCs of various origins (Fig. 2A). When the performance of the diagnostic tool was compared in a pairwise way, it showed a much higher accuracy, precision, recall, and F1 score in differentiating between OSCC and oesophageal SCC and cervical SCC than it did when it was compared in a multiwise fashion, suggesting the possibility that this model could be utilised as a diagnostic tool. However, the comparison considering OSCC and non-oral HNSCC exhibited much lower test accuracy as well as other factors, including test precision and training accuracy, suggesting the difficulty of differentiating OSCC from non-oral HNSCC (Fig. 2B). The authors suspect that the problem may be related to the close spatial relationship between the two types of tumours. To define factors responsible for this difference, the performance of the tool in distinguishing oesophageal SCC from oesophageal adenocarcinoma, which is another common cancer in the oesophagus, was analysed. The tool displayed low performance (~ 58%) in distinguishing specific types of oesophageal cancers, whereas the tool showed high discriminatory performance in comparisons of oesophageal SCC with other SCCs even though these SCCs resemble each other histopathologically. In addition, the tool exhibited high performance in discriminating non-oral HNSCC from SCCs of other tissue origins (Fig. 3). The findings suggest that the tool shows better performance in diagnosing cancers of different tissue origins than in discriminating histopathologically different cancers that are derived from the same type of tissue.

### Analysis of the diagnostic tool using machine learning classification suggests that a differential pattern of gene expression is a more important factor than histopathologic features for differentiating cancer types

**Fig. 4:**
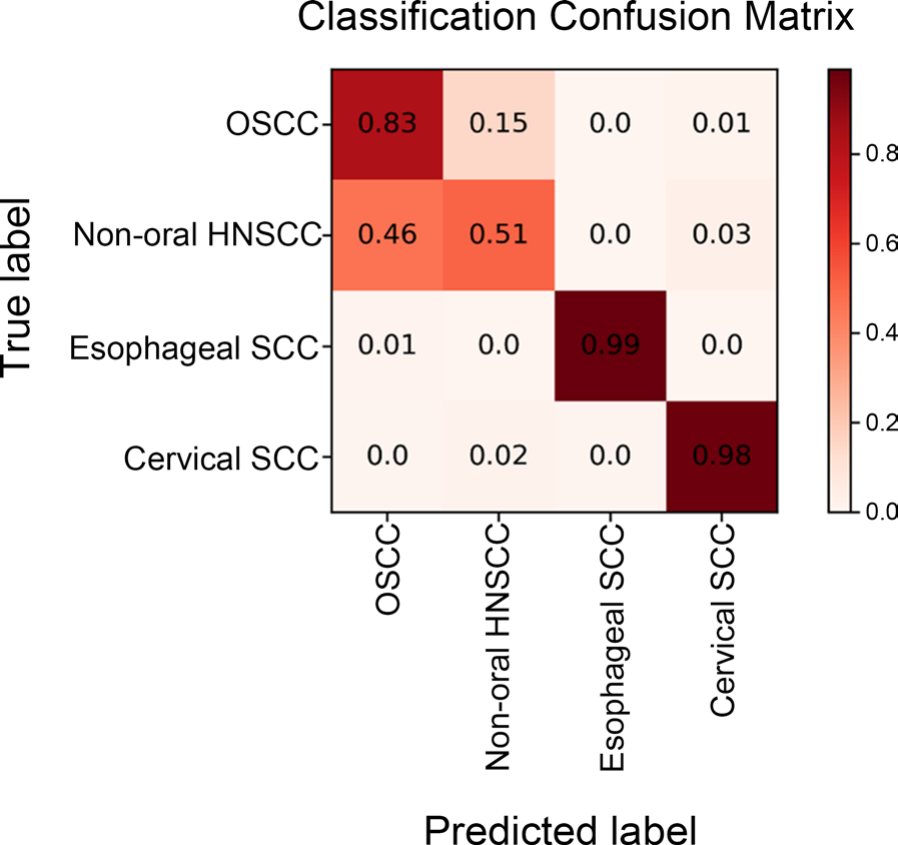
The confusion matrix for classification of SCCs

To clearly determine diagnostic accuracy in differentiating OSCC from SCC of other tissues or organs, a confusion matrix was generated, as shown in Fig. 4. Oesophageal and cervical SCCs were classified correctly at accuracies of 99% and 98%, respectively, and 83% of OSCC was also correctly diagnosed. However, more than half of the non-oral HNSCCs were misclassified as OSCC, and 15% of OSCC were misclassified as non-oral HNSCC, further confirming the lower efficiency in discriminating OSCC from non-oral HNSCC (Fig. 4).

**Fig. 5:**
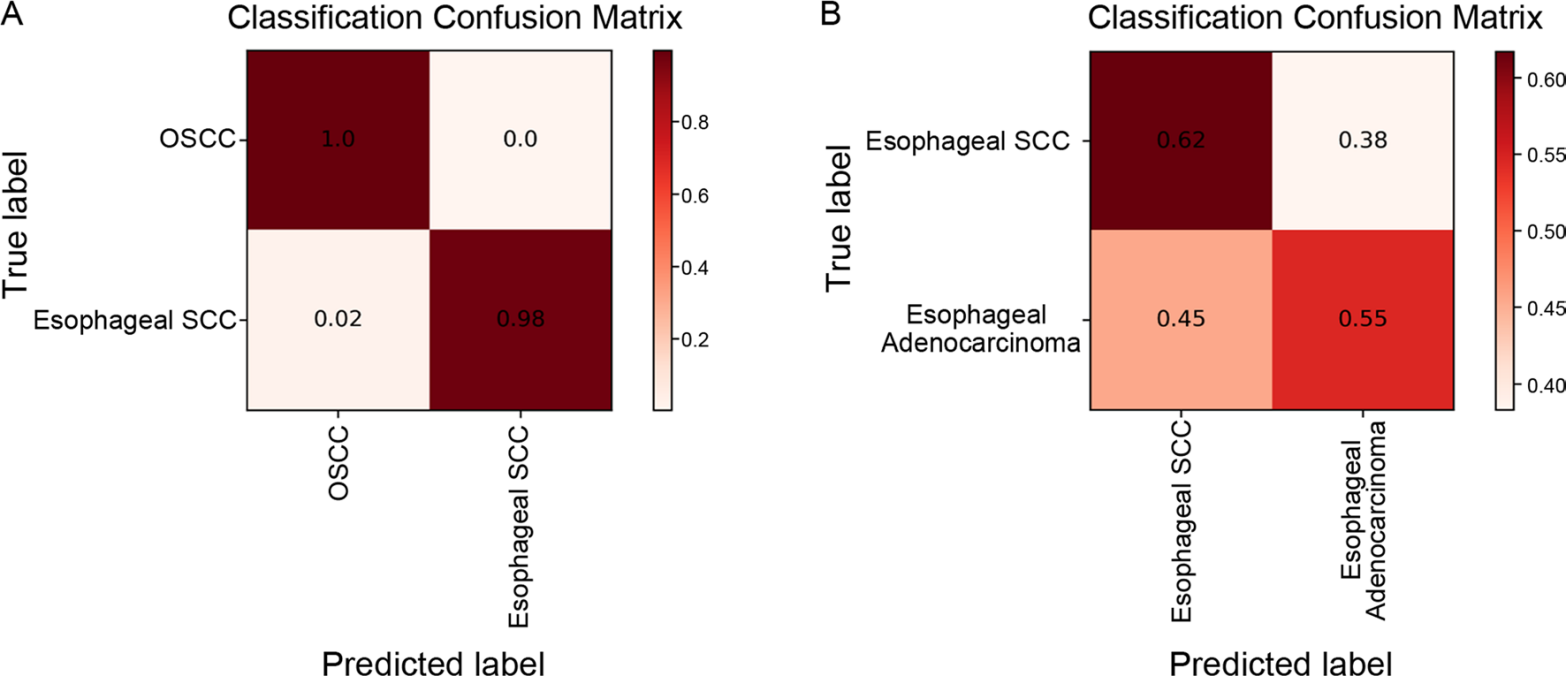
The confusion matrix for differentiation diagnosis of OSCC from oesophageal SCC (**A**) and of oesophageal cancers (**B**)

To further clarify the importance of the origin of cancer cells, the set of differentially expressed genes between oesophageal squamous cell carcinoma (ESCC) and oesophageal adenocarcinoma was compared to that between OSCC and ESCC. Although both OSCC and ESCC share very similar histopathologic features, the machine learning tool differentiated them easily, with only ~ 2% error when discriminating ESCC from OSCC, whereas 38% of ESCCs were misdiagnosed as oesophageal adenocarcinoma (Fig. 5A, B), implying that the gene expression pattern depends more on the site of tumour occurrence than the histopathologic identity and thus that tissue origin is a more important factor in determining tumour characteristics.

To identify valuable genes for diagnosing OSCC, the dominant genes that could discriminate OSCC from other SCCs were extracted through the class activation map feature extraction method in a multiwise manner. In addition, data on the characteristic genes of OSCC were extracted by individually comparing OSCC with other types of SCC in a pairwise manner: comparison (1) between OSCC and non-oral HNSCC, (2) OSCC and cervical SCC, and (3) OSCC and ESCC. Then, the shared genes from each comparison were selected (Table 1). We compared those genes from the extraction methods and found no consistent genes.

**Table 1:** List of 20 leader genes of OSCC extracted from machine learning tumour classification

No.	Gene name	
	Multi-wise	Pair-wise
1	KCNA10	FGF20
2	FOSL2	DLC1
3	PRDM16	ZNF705D
4	LUZP1	TUSC3
5	PAPOLG	SLC7A2
6	SIPA1L2	LGI3
7	TEX261	NUDT18
8	BCL10	MTUS1
9	NBPF14	PIWIL2
10	LAMC1	STC1
11	ERRFI1	CDCA2
12	PRAMEF20	DOCK5
13	FMO2	TIMM8A
14	GPA33	CENPI
15	PLA2G5	TSPAN6
16	TMEM167B	ARMCX6
17	GRIK3	TCEAL7
18	RPTN	TCEAL5
19	GPBP1L1	NXF3
20	NHLH1	TCEAL6

## Discussion

In the present study, we evaluated whether a diagnostic model based on CNN and visualisation methods using RNA sequencing data were useful in correctly recognising OSCC. We observed that there were no problems in differentiating OSCC from oesophageal or cervical SCCs, implying that OSCCs exhibit distinct gene expression from other SCCs and that histopathologic features cannot correctly reflect the genetic features of cancer cells. However, discrimination of OSCC from non-oral HNSCC was very difficult, and the discriminative ability of the model increased as distance from oral mucosa increased. Although it used a different data processing from ours, a previous study on molecular diagnostic methods using whole transcriptome RNA sequencing data from TCGA showed that oesophageal carcinomas were misclassified as stomach cancers. Their model also reported problems in distinguishing cervical cancers of different histopathologic types, such as SCC, adenosquamous carcinoma, and adenocarcinoma [11]. In this study, we also observed a similar limitation in differential diagnosis of different oesophageal cancers; for example, distinguishing SCC from adenocarcinoma with completely different morphologic features. It appears that genetic variations of cancer cells are site-specific, presumably with similar genetic background shared by the same site of origin. Accordingly, the intrinsic property of our data processing algorithm did not allow us to precisely discriminate cancers with different histopathologic findings once they are originated from the same tissue. While this limitation may restrict the efficacy of our computational analysis and needs to be improved for clinical applications, it could also validate the effectiveness of our system in detecting site-specific genetic variations. Together with a successful differential diagnosis of OSCC from oesophageal or cervical SCCs, these data provide further support to the power of our approach in improving diagnostic accuracy based upon genetic information. It should be noted that similar histopathologic features at a macroscopic level could develop from diverse arrays of genetic alterations at a molecular level. With this regard, it is crucial to dissect precise genetic programs underlying macroscopically similar histologic features to fully understand the pathophysiology of individual cancers. Therefore, information on tissue origin-based genetic variations could significantly contribute to successful implementation of cancer treatment remedies that mainly relies on complete comprehension of genetic changes and subsequent identification of optimized therapeutic targets.

Through the process of computational analysis for developing diagnostic models in this study, we were able to identify a set of genes with differential expression relevant to SCCs. Interestingly, the lists of genes extracted with different analytical methods were mutually exclusive. For example, a single groupwise comparison among all types of SCCs indicated *KCNA10*, *FOSL2*, and *PRDM16* as the top 3 candidates useful for the diagnosis of OSCC, whereas *FGF20*, *DLC1*, and *ZNF705D* were extracted as leader genes from pairwise comparisons. Furthermore, these two methods yielded non-overlapping sets of the top 20 candidate genes characteristic of OSCC. It is plausible that the commonly shared genes in all SCC types are often excluded from the final list of candidates in a single-grouped analysis, leaving only those with differential expression to be further considered. In contrast, the candidates more relevant to the identity of affected tissues in each SCC appear more likely to be extracted from pairwise comparisons, in line with the significant influence of tissue proximity on the genomic profiles relevant for the diagnosis of various SCCs. These analysis-specific outcomes may introduce confusion in developing common diagnostic tools or therapeutic targets based upon bioinformatical analyses of widely variable control and tissue types. Such diversity of extracted candidate genes in a method-dependent manner thus need to be further investigated to develop optimized analytical means for genomic profiles specific to individual cancer, such as OSCC, with the help of biological and experimental studies in combination.

In addition, a previous study using the TCGA and Gene Expression Omnibus (GEO) databases reported 6 primitive biomarkers of OSCC (*GNA14*, *CMA1*, *DKK1*, *HOXC6*, *HCG22*, and *HOTTIP*) that were not found in our results [13]. This discrepancy may be due to differences in the targets being compared. The study of Huang et al. identified genes by comparing gene expression profiles of OSCC tissue and normal controls, whereas we extracted diagnostic markers of OSCC by analysing SCCs of other tissues, and the extracted genes in this study are being reported in this context for the first time. Among the identified genes, *PRDM16* and *DLC1* have been studied in various types of cancers, and the significance of these genes in OSCC has not been examined [23–26]. Furthermore, the roles of *KCNA10* and *ZNF705D* in the pathogenesis of cancer, including OSCC, have not been investigated, implying the novel insights gained from this study and new avenues for the diagnosis of and new treatment strategies for OSCC.

In summary, we implemented a computation approach to develop novel biomarkers for OSCC based upon site-specific genetic variations that occur during the process of cancer progression. Further validation of these biomarkers in both experimental and clinical settings will strengthen the power of this computation approach in improving the accuracy of diagnosis and the assessment of treatment outcome and prognosis, in addition to its aid in development of optimized therapeutic strategies.

## Supplementary Information

**Additional file 1.** Numbers and types of tumor samples used in this study.

## Data Availability

The data used for this study comes from The Cancer Genome Atlas database. Detailed information and further explanation about the data source is provided on https://www.cancer.gov/about-nci/organization/ccg/research/structural-genomics/tcga. Dataset is publicly available on Broad Institute GDAC Firehose, can be accessed through https://gdac.broadinstitute.org/.
