# Supplementary material for: Authentication of differential gene expression in oral squamous cell carcinoma using machine learning applications
Source: BMC Oral Health. 2021 May 29;21:281. doi: 10.1186/s12903-021-01642-9 (PMC8164276; doi:10.1186/s12903-021-01642-9)
Supplement: Supplementary file 1 — Additional file 1. Numbers and types of tumor samples used in this study. [file 12903_2021_1642_MOESM1_ESM.docx]

**Supplementary Material**

TITLE:

Authentication of differential gene expression in oral squamous cell carcinoma using machine learning applications

Authors:

Rian Pratama, Jae Joon Hwang, Ji Hye Lee, Giltae Song, Hae Ryoun Park

Table S1. Numbers and types of tumor samples used in this study

| **Program** | **Subtype** | **Experimental Strategy** | **#Samples** |
| --- | --- | --- | --- |
| TCGA-HNSC | Oral Squamous Cell Carcinoma | RNA-Seq | 337 |
| TCGA-HNSC | Non-oral Squamous Cell Carcinoma | RNA-Seq | 182 |
| TCGA-CESC | Cervical Squamous Cell Carcinoma | RNA-Seq | 252 |
| TCGA-ESCA | Esophageal Squamous Cell Carcinoma | RNA-Seq | 88 |
| TCGA-ESCA | Esophageal Adenocarcinoma | RNA-Seq | 95 |
